# 1318. Clinical and Molecular Characteristics of Hypermucoviscous *Klebsiella pneumoniae* Causing Pneumonia in Korea

**DOI:** 10.1093/ofid/ofab466.1510

**Published:** 2021-12-04

**Authors:** Ji Yeon Lee, Hyun Ah Kim, Miri Hyun

**Affiliations:** Keimyung University School of Medicine, Daegu, Taegu-jikhalsi, Republic of Korea

## Abstract

**Background:**

Invasive *Klebsiella pneumoniae (K. pneumoniae*) was emerged in Asia, well-known for community-onset liver abscess. Healthcare-associated pneumonia caused by hypervirulent *K. pneumoniae* has been reported in recent studies. The purpose of this study was to evaluate the clinical and molecular characteristics of hypervirulent *K. pneumoniae* compared with classic *K. pneumoniae* in respiratory infection.

**Methods:**

The study was performed on 163 *K. pneumoniae* isolates of respiratory infections collected from Keimyung University of Dongsan Medical Center from November 2013 to November 2015; group A, as classic *K. pneumoniae* and group B, as hypervirulent *K. pneumoniae*. Hypermucoviscous phenotype was confirmed with string test. Capsular serotypes, *rmpA, magA, allS, mrkD, entB, kfu*, and *iutA* were identified using specific primers by polymerase chain reaction. The biofilm mass was determined using the microtiter plate assay measured by optical density (OD, 570nm).

**Results:**

A total 163 patients were analyzed, 100 (61.3%) of group A and 68 (38.7%) of group B. Community-acquired pneumonia was observed in 49.2% of group B and 18.0% of group A (*p*=0.001). Underlying diseases except chronic lung disease were more associated with group A. Mean age (72.6±11.7 vs. 68.8±12.5 years, *p*=0.051) and antimicrobial resistant rates were higher in group A. Mechanical ventilators (21.0% vs. 36.5%, *p*=0.030) was more associated with group B. Concordances of initial antibiotics (57.5% vs. 92.1%, *p*=0.001) were more observed in group B. Biofilm formation and infection related 30-day mortality showed no differences between the two groups.

**Conclusion:**

Contrary to our expectations, hypervirulent *K. pneumoniae* was more associated with community-acquired pneumonia in this study. Compared to classic *K. pneumoniae*, hypervirulent *K. pneumoniae* showed more association with severe pneumonia and less association with underlying diseases. In respiratory infection, biofilm formation was not different according to hypermucoviscousity.

**Disclosures:**

**All Authors**: No reported disclosures

